# Metabolomic predictors of phenotypic traits can replace and complement measured clinical variables in population-scale expression profiling studies

**DOI:** 10.1186/s12864-022-08771-7

**Published:** 2022-07-31

**Authors:** Anna Niehues, Daniele Bizzarri, Marcel J.T. Reinders, P. Eline Slagboom, Alain J. van Gool, Erik B. van den Akker, BBMRI-NL BIOS consortium, BBMRI-NL Metabolomics consortium, Peter A.C. ’t Hoen

**Affiliations:** 1 Center for Molecular and Biomolecular Informatics, Radboud Institute for Molecular Life Sciences, Radboud university medical center, Geert Grooteplein Zuid 26-28, Nijmegen, 6525 GA Netherlands; 2 Translational Metabolic Laboratory, Department Laboratory Medicine, Radboud university medical center, Geert Grooteplein Zuid 10, Nijmegen, 6525 GA Netherlands; 3 Molecular Epidemiology, LUMC, Einthovenweg 20, Leiden, 2333 ZC Netherlands; 4 Leiden Computational Biology Center, LUMC, Einthovenweg 20, Leiden, 2333 ZC Netherlands; 5 Delft Bioinformatics Lab, TU Delft, Van Mourik Broekmanweg 6, Delft, 2628 XE Netherlands; 6 Max Planck Institute for the Biology of Ageing, Cologne, Germany

**Keywords:** Multi-omics, Metabolomics, Transcriptomics, Surrogates, Predictors, Expression profiling, Population cohort study, Meta-analysis, Clinical surrogates, Surrogate outcomes

## Abstract

Population-scale expression profiling studies can provide valuable insights into biological and disease-underlying mechanisms. The availability of phenotypic traits is essential for studying clinical effects. Therefore, missing, incomplete, or inaccurate phenotypic information can make analyses challenging and prevent RNA-seq or other omics data to be reused. A possible solution are predictors that infer clinical or behavioral phenotypic traits from molecular data. While such predictors have been developed based on different omics data types and are being applied in various studies, metabolomics-based surrogates are less commonly used than predictors based on DNA methylation profiles.In this study, we inferred 17 traits, including diabetes status and exposure to lipid medication, using previously trained metabolomic predictors. We evaluated whether these metabolomic surrogates can be used as an alternative to reported information for studying the respective phenotypes using expression profiling data of four population cohorts. For the majority of the 17 traits, the metabolomic surrogates performed similarly to the reported phenotypes in terms of effect sizes, number of significant associations, replication rates, and significantly enriched pathways.The application of metabolomics-derived surrogate outcomes opens new possibilities for reuse of multi-omics data sets. In studies where availability of clinical metadata is limited, missing or incomplete information can be complemented by these surrogates, thereby increasing the size of available data sets. Additionally, the availability of such surrogates could be used to correct for potential biological confounding. In the future, it would be interesting to further investigate the use of molecular predictors across different omics types and cohorts.

**Supplementary Information:**

The online version contains supplementary material available at (10.1186/s12864-022-08771-7).

## Background

Genome-wide association studies (GWAS) have proven to be valuable in uncovering links between genes and a wide range of phenotypic traits. Such findings have led to the discovery of new disease-related biomarkers and are often the basis for gaining a better understanding of biological processes or disease-mechanisms. Since the introduction of the first GWAS powered by the availability of genome-wide single-nucleotide polymorphism (SNP) profiling, numerous studies have identified thousands of SNP-trait associations [1]. Technological advancements allowing high-throughput profiling of other molecular features, such as transcripts, or DNA methylation sites, also enabled population-scale studies of transcriptomics, epigenomics, and other omics data types.

Such studies are susceptible to confounding by biological and technical factors that can influence omics profiles and phenotypic traits of interest. However, measured values to correct for such confounding are often not available. As a solution, differences in cell type composition are commonly accounted for using information contained in the DNA methylation profiles themselves, by either reference-based imputation [2] or reference-free methods such as surrogate variable analysis (methods reviewed in [3] and [4]). Other well-known examples of inferring values for possible confounding factors from DNA methylation profiles include sex [5] and smoking status prediction. Bollepalli et al. [6] trained a smoking status classifier using multinomial LASSO regression. Machine learning approaches have also been applied to other omics data types to predict environmental exposures [7].

The value of such predictors is not only evident when complementing missing data to account for technical or biological confounding, but also for using them as outcome variables. These molecular surrogates can be used in association studies in order to link molecular features to clinical phenotypes or exposures. Since identified associations in ome-wide association studies often have only moderate effect sizes, a common approach to detect relevant features are cross-cohort meta-analyses [8]. However, the applicability of meta-analyses can be limited by availability of the respective outcomes of interest. Specific clinical, environmental, or phenotypic traits might not be recorded in every cohort, or the data collection might be based on different protocols, making the reported values for these traits not directly comparable.

As more and more multi-omics data sets become available, it becomes possible to make use of molecular predictors to infer phenotypic traits from specific omics layers. Blood is a key specimen in clinical diagnostics reflecting on the health state of an individual. While blood metabolomics methods partially overlap with classical clinical diagnostics methods, they can measure a wide range of metabolites and have the potential to play an important role in personalized medicine approaches [9]. Recently, Bizzarri et al. [10] trained predictors on proton NMR-based metabolomics (Nightingale Health) data. The authors applied logistic regression using elastic net regularization to train models for various clinical variables, including physiological measures, environmental exposures, and clinical endpoints. They demonstrated the use of these surrogates in metabolome-wide association studies to complement missing clinical data and correct for confounding. They further showed that metabolomic surrogates can help explore independent risk factors of all-cause mortality in older individuals [10].

We here propose the use of metabolomics-derived surrogates in analyses of other omics layers, not only as covariates to account for confounding factors, but also as outcome variables. We investigated whether values derived from molecular predictors represent a viable alternative to measured or reported clinical or phenotypic traits to serve as outcome variables in population-scale gene expression profile association studies. To this end, we applied 17 metabolomic predictors to metabolomics data from four large population cohorts inferring values for phenotypic, exposure, and clinical traits. We performed association studies on corresponding RNA-seq data sets employing either reported/measured or inferred values as outcome variables, and systematically compared the respective results of these analyses. For five of the outcomes, where reported values were not available, we evaluated the performance of the metabolomic surrogates based on results reported in literature.

## Results

**Fig. 1:**
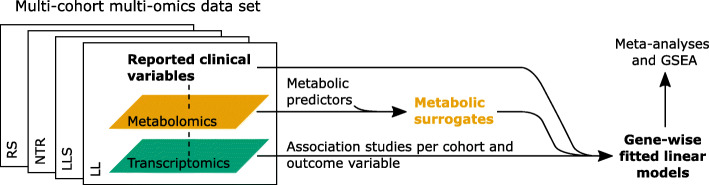
Overview of study workflow. Gene-wise models are fitted for various outcome variables based on reported information or metabolomic surrogates, respectively

In this study, we performed expression profiling studies to evaluate the performance of 17 surrogate outcomes that are based on molecular predictors. Metabolomics data used to infer surrogate outcome values and RNA-seq data used for the evaluation are part of multi-omics data sets of four large Dutch population cohorts: LifeLines (LL) [11], Leiden Longevity Study (LLS) [12], Netherlands Twin Register (NTR) [13], and Rotterdam Study (RS) [14]. An overview of the study workflow is shown in Fig. 1. For the different outcomes (Table 1), we compared expression profile association study results based on metabolomic surrogate outcomes to those based on reported outcomes whenever possible. Additionally, results were compared to literature findings.

**Table 1:** Overview of phenotypic traits. Availability of variable is indicated by ‘x’

Phenotypic trait	Reported outcome	Surrogate outcome
Low estimated Glomerular Filtration Rate (eGFR)		x
High triglycerides	x	x
High LDL-associated cholesterol	x	x
High total cholesterol	x	x
Low HDL-associated cholesterol	x	x
Diabetes		x
Metabolic syndrome		x
Sex	x	x
Lipid medication	x	x
BMI/obesity status	x	x
High high-sensitivity C-reactive protein (hsCRP)	x	x
Blood pressure lowering medication		x
Low hemoglobin	x	x
Low white blood cells	x	x
Current smoking	x	x
Alcohol consumption		x
High age (≥65 y.o.)	x	x

### Metabolomic surrogate outcomes

We inferred values for clinical variables by applying molecular predictors to metabolomics data. Recently reported metabolomic predictors trained on up to 22 Dutch population cohorts [10] were applied to infer values for outcome variables (Table 1). All 17 metabolomic predictors had been shown to perform accurately with mean AUC values >0.7 in a 5-fold cross-validation approach [10]. In order to avoid emphasis on clinical extremes, the metabolomic predictors trained by Bizzarri et al. are based on binary representations using clinical thresholds for continuous variables. The values returned by the predictors are continuous posterior probability scores for belonging to one of the two groups. In most cases, this is the clinical risk group. We here used these predicted values as surrogate outcomes and compared their use in expression profiling studies to reported or measured outcome values.

### Expression profile association studies

In the next step, the 17 metabolomic surrogates were used as outcomes of expression profile association studies. In addition to analyses employing these surrogate outcomes, analyses using measured or reported outcome values were performed. For five outcomes that had limited availability of reported values (eGFR, diabetes, metabolic syndrome, blood pressure lowering medication, and alcohol consumption) only surrogate outcomes were used. In each linear regression model, known biological (age, sex) and technical (flow cell number, white blood cell composition) confounding factors were included (formulas available in Additional file 1).

**Fig. 2:**
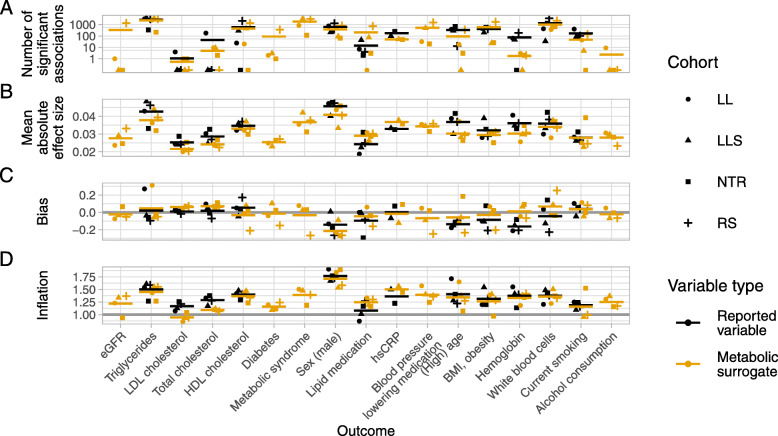
Comparison of association study result characteristics. Number of significant associations (based on bacon-corrected and FDR-corrected *p*-values $p_{b_{adj}}<0.05$) (A), mean absolute effect sizes (based on bacon-corrected effect sizes) across all genes (B), and bias (C) and inflation (D) of test statistics (*t*-statistic) for alternative models per comparisons and cohort. Horizontal lines for test-statistic mean =0 and standard deviation =1 of theoretical null distribution were added. Comparisons are ordered by performance of metabolomic predictors for binary outcome measures. Type of outcome variable is indicated by color: reported or measured variable = black, metabolomic surrogate = orange. Mean values across four cohorts (two cohorts for hsCRP) are plotted as horizontal bars. Note the log10 scale on the y-axis of the upper plot

For an initial assessment of the performance of each model, we compared the numbers of significant associations and the effect sizes between outcomes and outcome variable types. Additionally, test statistic (*t*-statistic) bias and inflation were estimated as parameters (mean and standard deviation) of the empirical null distribution using a Bayesian method implemented in the R package bacon [15] (Fig. 2). Numbers of identified significant associations (Fig. 2A) varied strongly across outcomes. Highest numbers were found for the outcomes triglycerides, metabolic syndrome, and white blood cells. For several outcomes, including eGFR, LDL cholesterol, total cholesterol, and alcohol consumption, no or only few significant gene-trait associations were found. For outcomes where association study results based on surrogate outcomes could be compared to results for reported variables, the numbers of identified significant associations averaged across all cohorts were higher for the metabolomic surrogate outcomes in two cases, and lower in 10 cases. However, the variation across the four cohorts was generally higher than the difference between models employing either reported or surrogate outcome variables. Similarly, high variation across cohorts was observed for the other parameters assessed to evaluate the performance of the models. Absolute effect size averaged across all genes (Fig. 2B) were generally small, with the highest values observed for the outcomes triglycerides and sex. In 10 cases, the mean absolute effect size averaged across cohorts was lower when using metabolomic surrogate outcomes instead of reported variables; in two cases, it was higher. We observed relatively low test statistic bias (Fig. 2C) across all outcomes and types of outcome variables. The bias, i.e., the deviation of the empirical null distribution’s mean from zero, averaged across cohorts decreased in four cases, increased in three cases, and remained similar in five cases when employing metabolomic surrogate outcomes instead of reported variables. Bias in the RS cohort was often higher than in the other cohorts. This may be explained with the differences in population structure. The RS cohort has a higher average age than the other three cohorts [16], indicating higher bias for the studied clinical variables in older populations. Inflation (deviation of the empirical null distribution’s standard deviation from one, Fig. 2D) was highest for the outcome sex. In most cases, inflation averaged across cohorts remained stable when using different outcome variable types. For the outcome total cholesterol, it slightly decreased when using metabolomic surrogate outcomes instead of reported variables; for the outcomes lipid medication and hsCRP, it slightly increased.

**Fig. 3:**
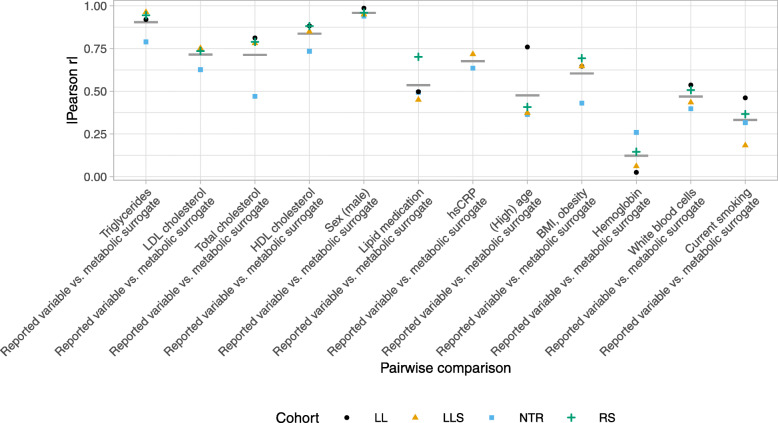
Pairwise comparisons of association study results. Absolute Pearson correlation coefficients (Pearson *r*) of bacon-adjusted regression coefficients of gene-wise linear models (limma/voom) for outcome variables in alternative models per comparison and cohort. Comparisons are ordered by performance (AUC) of metabolomic predictors for binary outcome measures. Mean values across four cohorts (two cohorts for hsCRP) are plotted as horizontal bars (gray)

Since the number of significant associations and average effect sizes do not allow drawing conclusions about the similarity of the association study results employing different types of variables as outcome, we next performed pairwise comparisons of models with different types of outcome variables, i.e., reported vs. surrogate. Figure 3 shows the correlation of regression coefficients from gene-wise fitted linear models between two different types of outcome variables. Correlation coefficients were generally high for surrogate outcomes based on best-performing metabolomic predictors. For outcomes based on predictors with reported AUC >0.9 (triglycerides, LDL cholesterol, total cholesterol, HDL cholesterol, and sex) [10], the correlation coefficients averaged across cohorts ranged between 0.71 and 0.96. We observed a modest trend for decreasing correlations with decreasing performance of predictors. However, there were exceptions, with sex having the highest correlation values, although the predictor’s AUC was reported to be lower than those for triglycerides or cholesterol. Lowest similarity of results with average absolute Pearson *r*<0.5 were observed for the outcomes age, white blood cells, smoking, and hemoglobin, the latter having the lowest correlation values. We often observed that correlations were lower for the NTR cohort. This could be explained by a technical difference in the metabolomic profiles, with NTR missing glutamine [10].

### Meta-analyses and replication studies

**Fig. 4:**
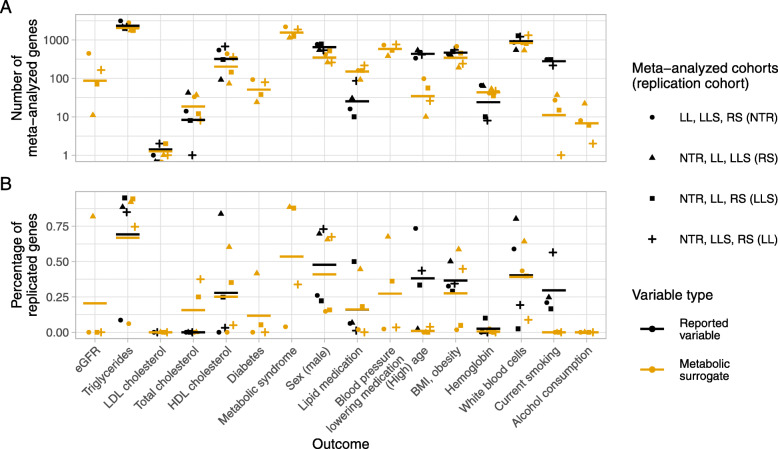
Meta-analyses and replication studies. Number of meta-analyzed genes (significant associations, bacon-adjusted *p*-values FDR-adjusted for multiple testing, *p*<0.05) in leave-one-cohort meta-analyses (A) and percentage of genes replicated (significant associations, Bonferroni-adjusted for multiple testing, *p*<0.05) in replication cohort (B). Type of outcome variable is indicated by color: reported or measured variable = black, metabolomic surrogate = orange. Mean values across four meta-analyses/replication studies are plotted as horizontal bars. Note the log10 scale on the y-axis of the upper plot

When comparing two alternative outcome variables, a lower or higher number of found significant associations does not necessarily imply that results are better or worse, since the values alone do not indicate if this is due to a reduction or increase of false positive (noise) or true positive findings, respectively. We observed that the expression profile association study results differ when surrogate values differ from reported values. However, we do not know which set of results is correct, as reported values could contain inaccuracies. Under the assumption that true positive findings, as opposed to false positive results, can be replicated in different cohorts (validating the results), we performed replication studies to determine which outcome variable type is more consistently reflected in the RNA-seq data. We performed leave-one-cohort-out meta-analyses and replication studies for all comparisons (except for hsCRP where only two cohorts were available) using the approach described by van Rooij et al. [16]. For each comparison, four meta-analyses were performed leaving one cohort out each time, and using the left out cohort for a replication analysis. Figure 4 shows the numbers of significant associations found in each meta-analysis (number of meta-analyzed genes) and the respective percentage of replicated genes. Overall, we did not find substantial differences in the numbers of meta-analyzed genes (Fig. 4A) except for the outcomes lipid medication, (high) age, and current smoking. While more genes were meta-analyzed when using the metabolomic surrogate for lipid medication, the reported variable yielded more meta-analyzed genes for age and smoking. For the latter two outcomes, results based on metabolomic surrogates could not be replicated (Fig. 4B) while on average 30-38% of results based on reported outcomes could be replicated. For these two outcomes we had also observed the highest differences between number of significations associations (Fig. 2A). For other outcome variables, the percentage of replicated genes was quite similar between outcome variable types, but the cohort which was left out for the meta-analysis had a strong impact on the results. Highest average replication rates were observed for triglycerides with 69% for the reported and 67% for the surrogate outcome. For a number of outcomes, associations could hardly be replicated: LDL-associated cholesterol, hemoglobin, and alcohol consumption. This is in line with the fact that almost no significant associations were found for these outcomes (except for reported hemoglobin) in the individual cohorts (Fig. 2).

### Gene set enrichment analysis

**Fig. 5:**
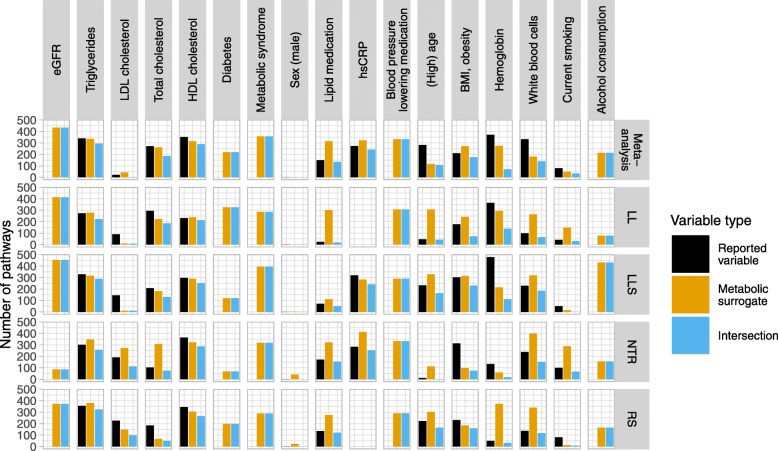
Gene-set enrichment analyses of association study results. Numbers of significantly enriched (Bonferroni-adjusted *p*<0.05) pathways (Reactome) for each outcome found in each cohort and in meta-analysis of all four (two for hsCRP) cohorts (top). Values for each type of outcome variable are represented as colored bars: reported variable = black, metabolomic surrogate = orange, intersection, i.e., pathways found by all outcome variables = blue

To arrive at a biological interpretation of the association study results, we performed gene-set enrichment analyses (GSEA) using pathways from the Reactome database. GSEA was applied to both individual cohort results and results from a meta-analysis of all four cohorts (Fig. 5). For direct comparisons of reported variables and metabolomic surrogates, we observed a highest overlap of significantly enriched pathways for HDL cholesterol and triglycerides in all cohorts, with 70-84% (meta-analysis 76%) and 65-80% (meta-analysis 77%) of significantly enriched pathways found by both reported and surrogate outcome, respectively. The overlap for the outcomes total cholesterol, lipid medication, hsCRP, BMI/obesity, and white blood cells was more variable across cohorts. Meta-analyzed results had an overlap of 38-69% and the order of significantly enriched pathways was highly comparable (see Additional file 2). The results for high age, current smoking, hemoglobin, and LDL cholesterol demonstrated a lower overlap than other outcomes and showed higher variation across the four cohorts compared to other outcomes. This is partially in line with the comparison of gene-wise linear models from association studies (Fig. 3), which showed that results for the outcome hemoglobin based on reported and inferred values were not correlated, and high age and current smoking were only moderately correlated. Since hardly any significant associations were found for hemoglobin (see Fig. 2A), the observed signal for this outcome was generally very low in the studied cohorts, independent of the type of outcome variable. It is surprising to observe that almost no significantly enriched pathways were observed for the outcome sex, even though many gene-trait associations were found and could be replicated in the meta-analysis and replication approach.

In order to evaluate the performance of surrogates for which results could not be compared to results based on reported outcomes, we compared significantly enriched pathways (see Additional file 2) to the literature. The top-ranked enriched pathways for low eGFR are related to translation. Since low eGFR is an indicator of kidney disease, this is in line with studies reporting increased translational activity to several kidney diseases [17, 18]. For diabetes and metabolic syndrome, which is a risk factor for diabetes, 16 out of the top 20 significantly enriched pathways were found for both outcomes. These include pathways related to translation, signaling, infection, and amino acid deficiency and metabolism. This is in agreement with previously reported results [19, 20]. For alcohol consumption, although almost no significant associations were found in individual analyses, several significant gene-outcome associations were found when meta-analyzing multiple cohorts (compare Figs. 2 and 4). Top-ranked positively enriched Reactome pathways from gene-set enrichment analysis (Additional file 2), including, e.g., innate immune system, signal transduction, and infectious disease, have been linked previously to chronic alcohol drinking [21].

## Discussion

While certain omics predictors especially based on DNA methylation profiles [2–5] are regularly applied in (multi) omics data analyses, metabolomics-based predictors are not commonly used in the analysis of other omics data types. Previously, Bizzarri et al. had shown that metabolomic surrogates can be used to correct for confounding in metabolome-wide association studies [10]. In this study, we investigated the use of these surrogates as outcome variables in the analysis of another omics level. We systematically compared results of population-scale gene expression profile association studies against outcome variables that were either reported or inferred by molecular predictors. The results generally showed good agreement (Fig. 2). Most similar association study results across all assessment parameters are those for the outcomes triglycerides and HDL cholesterol. Many significant gene-trait associations were found, of which many could be replicated, and the majority of significantly enriched pathways were found by reported and surrogate outcomes. Regression coefficients of the models including the reported and surrogate outcomes were strongly correlated, and the majority of pathways found by GSEA were obtained by both outcome types. The top-ranked pathways positively enriched for both high triglycerides and low HDL-cholesterol include “GTP hydrolysis and joining of the 60S ribosomal subunit”, “L13a-mediated translational silencing of Ceruloplasmin expression” and “Formation of a pool of free 40S subunits” which participate in the Eukaryotic translation initiation [22] and were previously shown to be enriched in a high-cholesterol and high-fat diet induced hypercholesterolemic rat model [23]. The metabolomic predictors for these outcomes are directly related to metabolic markers measured on the Nightingale platform and had shown high performance (AUC ≥0.95) [10]. It is expected that results based on predicted outcomes will depend on the accuracy of the prediction. Accordingly, we observed a slightly lower correlation between reported outcomes and metabolomic surrogates for molecular predictors that were known to have lower accuracy (Fig. 3). In the association studies for lipid medication and BMI/obesity, the molecular predictors yielded even more significant gene-trait associations than the reported outcomes (Fig. 2A) and a higher number of significantly enriched pathways were obtained when using the metabolomic surrogates (Fig. 5). For lipid medication, this may be related to inaccurate recording of this trait in the questionnaires used. For BMI, this may be explained by the more direct capturing of metabolic processes that are associated with obesity as a combined measure of BMI and waist circumference, because BMI alone is not a perfect indicator of metabolic health [24]. Similarity of results based on surrogate and reported value for sex was high across all assessment parameters, but GSEA did not yield significantly enriched pathways in most cases. It is possible that the genes significantly associated with sex belong to too many pathways and/or that some genes within a pathway have a positive association while others have a negative association resulting in a failure to identify positively or negatively enriched pathways. For the outcomes total cholesterol and hsCRP, regression coefficients were moderately correlated and GSEA results were very similar. For the outcome white blood cells overlap of significantly enriched pathways in GSEA was smaller. However, the order of top-ranked pathways was similar (see Additional file 2). Additionally, the application of metabolomic surrogates for outcomes that were not reported in the data and the comparison of association studies and GSEA results with literature show that these surrogate outcomes allow transferring information from one data set (the training data) to another, thereby facilitating to study phenotypes or exposures in data sets which would otherwise not be possible. These results suggest that metabolomic surrogates are a useful tool to complement phenotypic information of multi-omics data sets and enable analyses of clinical outcomes even when they are not reported. This is especially useful when reanalyzing existing data sets. Even though this study comprises four different large population cohorts and 17 metabolomic surrogates, it will be interesting to investigate in the future whether similar results can be observed in other cohorts, for other clinical variables, or for other omics data types.

For both outcome variable types, only few gene-trait associations were significantly associated with the outcome LDL cholesterol. Similarly, low numbers of significant associations were observed for hemoglobin for the surrogate outcome. In contrast to that, the reported outcome yielded more associations which, however, could not be replicated. For high age and current smoking slightly fewer associations were found when using the surrogate outcomes. They also performed worse in the meta-analyses and replication studies compared to reported outcome values. Here, differences between results based on different outcome variable types are reflected in lower correlations of regression coefficients of the gene-wise models and in a smaller overlap in enriched pathways from GSEA. Possible reasons for the differences between surrogates and reported values are lower performance of the metabolomic predictors for these outcomes, or a lower biological signal for the respective clinical outcomes and thus increased noise in the studied data. It is known that aging is reflected in transcriptomics data [25], but the metabolomic predictor for high age trained on binarized data (≥65 y.o.) might not be an ideal surrogate to study this. Alternatively, a metabolomics-based biological age predictor based on continuous data [26] might perform better. Differences between GSEA results of different outcome variable types could also arise from different biological information captured by the metabolomic surrogates and by reported or measured values. This phenomenon is known from epigenetic clocks whose age predictions can differ from chronological age, and different clocks can reflect different aspects of biological age [27]). While many of the top-ranked pathways (Additional file 2) for smoking were found by both outcome variable types, some pathways were solely found by using either reported smoking status (“smoking_current”) or metabolomic surrogate(“s_current_smoking”). Several pathways related to translation initiation (“Formation of the ternary complex, and subsequently, the 43S complex”, “Translation initiation complex formation”, “Ribosomal scanning and start codon recognition”) were only significantly enriched when using the reported variable as outcome. Translation of mRNA is known to be dysregulated in cancers [28]. Pathways only enriched when using the metabolomic surrogate include “Platelet activation, signaling and aggregation” and “Hemostasis”. Increased platelet aggregation has been reported in smokers [29] and platelet-dependent thrombin levels were shown to be increased in smokers and following smoking [30]. This possibly indicates that the reported smoking behavior captures effects of long-term exposure to smoking better, while the metabolomic surrogate captures effects of acute smoking. It would be interesting to further investigate which aspects of the clinical phenotypes are captured by the metabolomic surrogates. This requires additional phenotypic information. To understand which aspect of smoking behavior is reflected in the omics data current smoking status alone might not be sufficient. More information including pack years and years since smoking cessation could help better understand the information captured by the predictors. It is also possible that different omics types capture different effects better, e.g., short-term and long-term effects. In that case, combining reported outcome variables, and/or molecular surrogates from different omics layers could be very useful, not only to study the effect of a certain exposure, but also to better adjust for confounding factors.

## Conclusions

In our systematic comparison of expression profiling results using either reported variables as outcome or surrogate outcomes inferred from metabolomics profiles, we demonstrated that in many cases metabolomic surrogates yield similar results as reported variables. We showed that the availability of these surrogate outcomes extends the possibilities of studying various clinical outcomes in population cohorts. It can enable the reuse of existing multi-omics data with limited reported clinical (meta)data. This allows for inclusion of more cohorts in meta-analyses, even when outcomes of interest were not reported for all cohorts. This approach also increases possibilities to study clinical outcomes by allowing to infer important confounding factors. Especially investigations that rely on reuse of existing data, e.g., in the case of rare disease studies which often also suffer from low sample sizes, will benefit from this approach.

## Methods

### Data

In this study, we analyzed RNA-seq and metabolomics data from four large Dutch population cohorts: LifeLines (LL) [11], Leiden Longevity Study (LLS) [12], Netherlands Twin Register (NTR) [13], and Rotterdam Study (RS) [14, 31]. The data is provided by the Dutch node of the European Biobanking and BioMolecular Resources and Research Infrastructure (BBMRI-NL).

RNA-seq data of all four cohorts was generated by the BBMRI-NL Biobank-based Integrative Omics Study (BIOS) Consortium at the Human Genotyping facility (HugeF) of ErasmusMC, the Netherlands. RNA sample processing and sequencing is described in detail by Zhernakova et al. [32]. Briefly, total RNA was extracted from whole blood, depleted of globin transcripts, and paired-end sequencing of 2x50-bp reads was conducted using the Illumina HiSeq 2000 platform. Read alignment to reference genome hg19 was performed using STAR (v2.3.0). We used the “Freeze2 unrelated data sets”, which contain maximum sets of unrelated individuals and are available within the BIOS workspace at the SURF Research Cloud via the R package BBMRIomics v3.4.2 [33].

Metabolomics data was generated by the BBMRI-NL Metabolomics Consortium in 2014 as described by van den Akker et al. [26]. Briefly, metabolite concentrations were measured in EDTA plasma by proton nuclear magnetic resonance (^1^H-NMR) spectroscopy on the platform of the Nightingale Health Group (Helsinki, Finland) [34].

### Data analysis

All analyses were implemented in an R v4.0.3 [35] workflow employing R packages renv v0.14.0 for package management and drake v7.13.2 for workflow management. The analyses were run in the BIOS workspace of the SURF Research Cloud which is part of the multi-omics analysis platform of BBMRI-NL. The code to run the analyses in available in GitHub [36] and archived in Zenodo [37].

### Data preprocessing

#### Normalization of values for clinical traits

Numeric values were used for all reported clinical variables. In case of categorical variables, they were binarized as follows. For smoking status, “current smoker” was coded as 1, and “former-smoker” and “non-smoker” were coded as 0; for sex, “male” and “female” were coded as 1 and 0, respectively; for lipid medication, “statins” were coded as 1, and “no” and “yes, but no statins” were coded as 0. In order to be able to compare effect sizes in association studies, all clinical variables were standardized to zero-mean and unit-variance (z-score normalization).

#### RNA-seq data preprocessing

Samples with more than 10% missing values in the RNA-seq data were excluded from the analysis. Additionally, for comparisons of models employing either reported or inferred values as outcome, samples missing reported values were excluded. Subsequently, features (transcripts) missing in more than 10% of the samples were removed from the data set. Number of retained samples and features are given in Additional file 1.

The RNA-seq read counts as provided by the BBMRIomics R package were then normalized and transformed based on a previous evaluation of analysis strategies [16] as follows. Scaling factors for library sizes were calculated using the trimmed mean of M-values (TMM) method [38] implemented in the R/Bioconductor package edgeR v3.32.1 [39]. Using these scaling factors to adjust for sequencing depth, counts were transformed to log2 counts-per-million (CPM) reads, their mean-variance relationships were estimated using voom [40] implemented in the R/Bioconductor package limma v3.46.0, and the associated observation-level weights were used in the subsequent linear modeling to adjust for heteroscedasticity.

#### Metabolomics data preprocessing

The Nightingale Health metabolomics features inquired are the 56 variables selected by van den Akker et al. [26]. Outliers were identified as the samples having more than 1 missing observation (301 removed), more than 1 data point under the detection limit (49 removed) and having a value more than 5 standard deviations away from the overall mean observed within BBMRI-NL (0 removed). Remaining with a total of 12926 samples (LLS = 2343, LL = 1475, RS = 5136, NTR = 3972). The remaining 4210 missing values (0.58% of the entire dataset) were imputed as zero and the metabolomic features were z-scaled using the mean and standard deviations observed in BBMRI-NL. The samples were further filtered based on availability of corresponding RNA-seq data. Sample sizes per cohort and model are listed in Additional file 1.

### Metabolomic surrogates

Seventeen logistic regression models trained on up to 22 cohorts, including LL and RS, were applied to the dataset as described in Bizzarri et al. [10]. The surrogates used in this study are the posterior probabilities which represent how likely an individual is at risk for each of the inquired common clinical variables.

### Expression profile association studies

To determine association of profiles with clinical outcomes, gene-wise linear regression models were fitted using limma [41]. Known potential biological (sex, age) and technical confounders (flow cell number, white blood cell composition) were included in the models. Association studies were performed separately for the respective type of outcome variable, i.e., reported variable or metabolomic surrogate. Parameters for each linear model are summarized in Additional file 1. We adjusted *p*-values and effect sizes for statistical bias and inflation using the Bayesian method bacon [15], which estimates bias and inflation as parameters from the empirical null distribution of test statistics (*t*-statistic). Additionally, *p*-values were adjusted for multiple testing using the false discovery rate (FDR) [42, 43]. Results for two different variable types for the same outcome, were compared by calculating Pearson correlation coefficients of regression coefficients from gene-wise fitted models.

### Meta-analysis

Leave-one-cohort-out meta-analyses and replication studies in left out cohort were performed as described in [16].

### GSEA

Gene-set enrichment analyses (GSEA) were performed using the R/Bioconductor package fgsea v1.16.0 [44] and gene sets retrieved from the Reactome Pathway Database [22]. Genes were ranked by − log10(*p*_*b*_)∗|*β*_*b*_| with *p*_*b*_ = bacon-corrected *p*-value and *beta*_*b*_ = bacon-corrected effect size. The number of permutations for initial estimation of *p*-values was set to 1×10^4^; the boundary for calculating *p*-values was set to 1×10^−50^.

## Supplementary Information

**Additional file 1** TWAS model parameters. This.csv file contains variable names, sample and feature numbers, and covariates per TWAS model and cohort.

**Additional file 2** Significantly enriched pathways from GSEA. This.html file contains plots showing significantly enriched pathways for each outcome variable. GSEAs are based on meta-analyzed TWAS results.

## Data Availability

The data underlying the results presented in the study are available from BBMRI-NL and access can be requested via the BIOS Consortium [47] and the BBMRI Metabolomics Consortium [48]. The code used to perform analyses for this study is available in GitHub [36] and archived in Zenodo [37].
