# Supplementary material for: Metabolomic predictors of phenotypic traits can replace and complement measured clinical variables in population-scale expression profiling studies
Source: BMC Genomics. 2022 Jul 31;23:546. doi: 10.1186/s12864-022-08771-7 (PMC9339202; doi:10.1186/s12864-022-08771-7)
Supplement: Supplementary file 2 — Additional file 2 Significantly enriched pathways from GSEA. This.html file contains plots showing significantly enriched pathways for each outcome variable. GSEAs are based on meta-analyzed TWAS results. [file 12864_2022_8771_MOESM2_ESM.html]

S1 Appendix


# S1 Appendix

#### Anna Niehues1

## 0.1 Results of gene set enrichment analyses

Gene-set enrichment analyses (GSEA) were performed using the R/Bioconductor package fgsea and gene sets retrieved from the Reactome Pathway Database . Genes were ranked by \(-\log\_{10}(p\_{b})\*|\beta\_{b}|\) with $p\_{b} $ = bacon-corrected p-value and \(beta\_{b}\) = bacon-corrected effect size. The number of permutations for initial estimation of p-values was set to ; the boundary for calculating p-values was set to .

## 0.2 eGFR

```
## Loading required package: dplyr
```

```
## Warning: package 'dplyr' was built under R version 4.0.5
```

```
## 
## Attaching package: 'dplyr'
```

```
## The following object is masked from 'package:gridExtra':
## 
##     combine
```

```
## The following object is masked from 'package:AnnotationDbi':
## 
##     select
```

```
## The following objects are masked from 'package:IRanges':
## 
##     collapse, desc, intersect, setdiff, slice, union
```

```
## The following objects are masked from 'package:S4Vectors':
## 
##     first, intersect, rename, setdiff, setequal, union
```

```
## The following object is masked from 'package:Biobase':
## 
##     combine
```

```
## The following objects are masked from 'package:BiocGenerics':
## 
##     combine, intersect, setdiff, union
```

```
## The following objects are masked from 'package:stats':
## 
##     filter, lag
```

```
## The following objects are masked from 'package:base':
## 
##     intersect, setdiff, setequal, union
```

Figure 1: GSEA results of meta-analyzed TWAS results for outcome eGFR (s\_low\_eGFR, metabolic surrogate).

## 0.3 Triglycerides

Figure 2: GSEA results of meta-analyzed TWAS results for outcome triglycerides (s\_high\_triglycerides, metabolic surrogate; triglycerides, reported variable).

## 0.4 LDL cholesterol

Figure 3: GSEA results of meta-analyzed TWAS results for outcome LDL cholesterol (s\_high\_ldl\_chol, metabolic surrogate; ldlchol, reported variable).

## 0.5 Total cholesterol

Figure 4: GSEA results of meta-analyzed TWAS results for outcome total cholesterol (s\_high\_totchol, metabolic surrogate; totchol, reported variable).

## 0.6 HDL cholesterol

Figure 5: GSEA results of meta-analyzed TWAS results for outcome HDL cholesterol (s\_low\_hdlchol, metabolic surrogate; hdlchol, reported variable).

## 0.7 Diabetes

Figure 6: GSEA results of meta-analyzed TWAS results for outcome diabetes (s\_diabetes, metabolic surrogate).

## 0.8 Metabolic syndrome

Figure 7: GSEA results of meta-analyzed TWAS results for outcome metabolic syndrome (s\_metabolic\_syndrome, metabolic surrogate).

## 0.9 Sex (male)

```
plot_pathways(readd(meta_GSEA_by_comparison_Sex..male_))
```

```
## [1] "No significantly enriched pathways."
```

## 0.10 Lipid medication

```
## Warning: Removed 1 rows containing missing values (geom_segment).
```

```
## Warning: Removed 1 rows containing missing values (geom_point).
```

Figure 8: GSEA results of meta-analyzed TWAS results for outcome lipid medication (s\_lipidmed, metabolic surrogate; lipidmed\_statins, reported variable).

## 0.11 hsCRP

Figure 9: GSEA results of meta-analyzed TWAS results for outcome hsCRP (s\_high\_hscrp, metabolic surrogate; hscrp, reported variable).

## 0.12 Blood pressure lowering medication

Figure 10: GSEA results of meta-analyzed TWAS results for outcome blood pressure lowering medication (s\_blood\_pressure\_lowering\_med, metabolic surrogate).

## 0.13 Age

Figure 11: GSEA results of meta-analyzed TWAS results for outcome age (s\_high\_age, metabolic surrogate; sampling\_age, reported variable).

## 0.14 BMI, obesity

Figure 12: GSEA results of meta-analyzed TWAS results for outcome BMI/obesity (s\_obesity, metabolic surrogate; bmi, reported variable).

## 0.15 Hemoglobin

Figure 13: GSEA results of meta-analyzed TWAS results for outcome hemoglobin (s\_low\_hgb, metabolic surrogate; hgb, reported variable).

## 0.16 White blood cells

Figure 14: GSEA results of meta-analyzed TWAS results for outcome white blood cells (s\_low\_wbc, metabolic surrogate; wbc, reported variable).

## 0.17 Current smoking

Figure 15: GSEA results of meta-analyzed TWAS results for outcome current smoking (s\_current\_smoking, metabolic surrogate; smoking\_current, reported variable).

## 0.18 Alcohol consumption

Figure 16: GSEA results of meta-analyzed TWAS results for outcome alcohol consumption (s\_alcohol\_consumption, metabolic surrogate).

---

1. Radboud University Medical Center, Anna.Niehues@radboudumc.nl↩︎
